# Reverse genetics through random mutagenesis in *Histoplasma capsulatum*

**DOI:** 10.1186/1471-2180-9-236

**Published:** 2009-11-17

**Authors:** Brian H Youseff, Julie A Dougherty, Chad A Rappleye

**Affiliations:** 1 Departments of Microbiology and Internal Medicine, The Center for Microbial Interface Biology, Ohio State University, 484 W. 12th Avenue, Columbus, OH 43210, USA

## Abstract

**Background:**

The dimorphic fungal pathogen *Histoplasma capsulatum *causes respiratory and systemic disease in humans and other mammals. Progress in understanding the mechanisms underlying the biology and the pathogenesis of *Histoplasma *has been hindered by a shortage of methodologies for mutating a gene of interest.

**Results:**

We describe a reverse genetics process that combines the random mutagenesis of *Agrobacterium*-mediated transformation with screening techniques to identify targeted gene disruptions in a collection of insertion mutants. Isolation of the desired mutant is accomplished by arraying individual clones from a pool and employing a PCR-addressing method. Application of this procedure facilitated the isolation of a *cbp1 *mutant in a North American type 2 strain, a *Histoplasma *strain recalcitrant to gene knock-outs through homologous recombination. Optimization of cryopreservation conditions allows pools of mutants to be banked for later analysis and recovery of targeted mutants.

**Conclusion:**

This methodology improves our ability to isolate mutants in targeted genes, thereby facilitating the molecular genetic analysis of *Histoplasma *biology. The procedures described are widely applicable to many fungal systems and will be of particular interest to those for which homologous recombination techniques are inefficient or do not currently exist.

## Background

The dimorphic fungal pathogen, *Histoplasma capsulatum*, parasitizes phagocytic cells of the mammalian immune system and causes one of the most common respiratory fungal infections world wide [1-3]. The mycelia-produced *Histoplasma *conidia are acquired by inhalation into the respiratory tract where exposure to mammalian body temperatures triggers their differentiation into pathogenic yeast cells [3,4]. *Histoplasma *virulence requires this transition to the yeast phase and expression of the corresponding yeast-phase regulon [5-7]. This transcriptional profile includes genes encoding specific factors that promote *Histoplasma *virulence [7-9]. While mammalian alveolar macrophages efficiently phagocytose *Histoplasma *cells, they are unable to kill the yeast [10-12]. Within the macrophage, *Histoplasma *modifies the intracellular compartment to promote its survival and replication. The ability to subvert immune defenses and to survive within phagocytes enables *Histoplasma *to cause disease in both immunocompromised and immunocompetent individuals. This high potential for infection is reflected in the fact that histoplasmosis is one of the most common pulmonary fungal infections among healthy individuals [13].

The mechanistic details that underlie *Histoplasma *pathogenesis are still largely unknown owing to limited or inefficient genetic methodologies. The genome sequences of three phylogenetically distinct strains of *Histoplasma *have been completed (North American type 1, NAm 1; North American type 2, NAm 2, and a lineage from Panama, Pan) [14] which has accelerated the ability to identify, define, and analyze *Histoplasma *genes. However, demonstration that a gene product contributes to a particular facet of biology requires specific depletion of the candidate factor and comparison to a factor-replete strain in functional tests. Targeted deletion of candidate factors is most often accomplished through genetic means, employing homologous recombination to replace the wild-type gene with an engineered deletion or disruption allele. In *Saccharomyces cerevisiae*, homologous recombination is so efficient that gene deletion libraries have been compiled with mutants representing entire sets of genes or even the majority of the genes in the genome [15,16]. In contrast, non-homologous or illegitimate recombination dominates in the dimorphic fungal pathogens [17], frustrating gene deletion attempts and impeding advancement of our molecular understanding of these fungi. Furthermore, *Histoplasma *can maintain introduced DNA (e.g. a deletion allele) as an extrachromosomal element which impedes efforts to incorporate alleles into the genome [18,19].

Despite these obstacles, genes have been deleted in *Histoplasma *following development of a two-step procedure [20]. Realization of the rare homologous recombination event necessitates a very large population as the frequency of allelic replacement is on the order of 1 in 1000 transformants [21]. As typical transformation frequencies are insufficient, individual transformants harboring recombination substrates are instead cultured and repeatedly passaged to generate a large number of potential recombination events. In the second step, a dual positive and negative selection scheme enriches the population for the desired recombinant. In practice, only a portion of the isolated clones harbor the deletion requiring screening of many potential isolates. In *Histoplasma*, this process of reverse genetics (the generation of a mutant in a targeted gene) has been successfully accomplished for only six genes to date, the vast majority in the Panama phylogenetic group (*URA5*, *CBP1*, *AGS1*, *AMY1*, *SID1*) [20-24]. For reasons not well understood, this procedure has not been very successful in the *Histoplasma *NAm 2 lineage despite numerous attempts. Recently, a deletion of the gene encoding DPPIVA has been reported in the NAm 2 lineage [25].

The inefficient and laborious process of deleting genes in *Histoplasma *prompted development of RNA interference (RNAi) as an alternative method to determine the role of gene products in *Histoplasma *biology [22]. To date, eight genes have been functionally defined by RNAi (*AGS1*, *UGP1*, *DRK1*, *YPS3*, *RYP1*, *GGT1 DPPIVA*, *DPPIVB*) [7,8,22,23,25-27]. However, RNAi can not generate a complete loss of function, and this potential for residual function imposes difficulties in interpreting negative results with RNAi (i.e. the absence of a phenotype). Unlike chromosomal mutations which are more permanent, plasmid-based RNAi effects must be constantly maintained with selection. For studies of *Histoplasma *virulence, this in vivo selection of plasmids requires use of the *URA5 *selection marker and *ura5 *auxotrophic *Histoplasma *strains.

To overcome these obstacles and limitations in our current techniques for genetic analysis of *Histoplasma*, we have developed a procedure for isolating chromosomally-located gene mutants without reliance on homologous recombination. We employed random mutagenesis to create collections of mutants. One approach to identify the desired gene disruption would be to characterize the mutation of each isolate in the collection of random mutants. However, this requires many resources, substantial time and effort, and thus is not well suited for studies targeting a particular gene. In forward genetics, random mutagenesis is successful because the desired mutant can be typically isolated or identified out of the much larger collection of mutants by growth phenotype or morphological changes. In reverse genetics, the mutant phenotype is the very aspect under investigation and thus mutants can not be identified by predicted changes. To enable reverse genetics following random mutagenesis in *Histoplasma*, we adapted PCR-based procedures employed for large scale screening in *Arabidopsis *and *C. elegans *[28-30]. We optimized a mutant pooling strategy and utilized PCR to efficiently identify mutant pools which contain the strain with the disrupted gene. To extract the strain with the targeted mutation, the pool is subsequently subdivided and individual clones addressed and screened by PCR. We demonstrate the effectiveness of this method by employing it to isolate a *cbp1 *mutant in the NAm 2 *Histoplasma *strain background.

## Results and Discussion

### Insertion mutant screening

**Figure 1:**
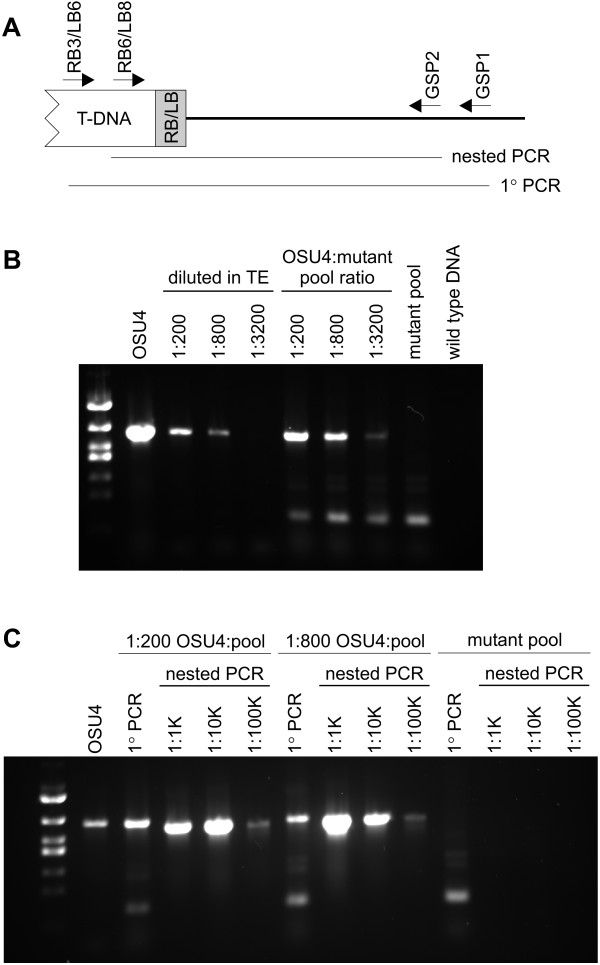
**Sensitivity of PCR to detect individual strains within pools of *Histoplasma *mutants**. Within a collection of *Histoplasma *yeast, PCR can identify cells comprising as little as 1/800^th ^of the population. **(A) **Schematic representation of the nested PCR screening approach for identification of T-DNA insertions in a targeted gene. Primers specific for the T-DNA left border (LB) or right border (RB) bind within the T-DNA element and gene specific primers (GSPs) anchor PCR from the chromosome. **(B) **Results of primary PCR experiments to detect the OSU4-specific T-DNA insertion. Template nucleic acid from OSU4 was diluted into TE buffer (1:200, 1:800, or 1:3200 dilutions) or template nucleic acid was prepared from suspensions of OSU4 yeast mixed with random T-DNA mutants at ratios of 1:200, 1:800, or 1:3200. Negative template controls consisted of wild-type Histoplasma DNA or nucleic acid prepared from the mutant pool before spiking with OSU4 yeast. Thirty cycles of PCR were performed using RB6 and AGS1-50 primers. The approximately 1250 bp amplicon is specific for the T-DNA insertion carried by the OSU4 strain. **(C) **Results of nested PCR performed on dilutions of the primary PCR from (B). 1:1000, 1:10,000, and 1:100,000 serial dilutions of the primary PCR reactions were used as templates for PCR with the nested primers RB6 and AGS1-72. PCR products were separated by electrophoresis through 1% agarose.

To generate insertion mutations in the *Histoplasma *genome, we used *Agrobacterium tumefaciens*-mediated transformation. This mutagen was selected because *Agrobacterium*-mediated transfer of T-DNA is efficient in producing random insertional mutations in *Histoplasma *yeast cells [23,31]. The majority of T-DNA insertions are single integration events [31] and thus the chance of secondary background mutations is minimized. Other mutagens such as UV or chemical agents result in multiple changes to the genome, and while these background mutations can be removed by repeated backcrossing of mutants to wild type, no reliable techniques for crossing laboratory strains have been developed for *Histoplasma *[32-34]. Additionally, insertional mutagens provide a molecular tag with known sequence (e.g. the T-DNA element) which we can exploit in PCR-based screening for mutations in particular chromosomal loci using a T-DNA specific primer in conjunction with a primer specific for the targeted gene (Figure 1A). The molecular weight of the PCR amplicon provides an estimate of the distance from the gene specific primer to the T-DNA insertion, and this distance can be used to determine whether the T-DNA element disrupts the targeted gene.

### Optimization of pool size for reliable detection of targeted mutations

As the successful isolation of a mutant in a targeted gene depends critically on the ability to identify a positive individual among a much larger population, we determined the PCR detection limit for different pool sizes. *Histoplasma *strain OSU4 harbors a T-DNA insertion in the *AGS1 *gene in which the T-DNA right border is oriented towards the 3' end of the *AGS1 *gene. Performance of PCR using a right border T-DNA primer and an *AGS1 *gene-specific primer produces a PCR amplicon of 1242 bp. To estimate the detection limit afforded by PCR in which a single strain could be found among a population of 200, 800, or 3200 mutants, 50 ng of nucleic acid purified from OSU 4 were diluted 1:200, 1:800, and 1:3200 with TE buffer and PCR performed on these templates with RB3 and AGS1-50 primers. With 30 cycles, PCR could consistently detect the OSU4 template when diluted as much as 1:800 (Figure 1B). To better approximate the condition where the desired mutant would be present among a much larger population of other T-DNA insertions, we mixed OSU4 with a pool of random T-DNA insertion mutants at a OSU4 yeast-to-mutant pool ratio of 1:200, 1:800, and 1:3200. Nucleic acids were purified from each pool and PCR was performed as before with 50 ng of total nucleic acid as templates. The positive 1242 bp amplicon was detected when OSU4 was present in as little as 1/800^th ^of the total population of yeast (Figure 1B). A faint band representing the *ags1*::T-DNA PCR product was observed when OSU4 constituted 1/3200^th ^of the template. No *ags1*::T-DNA PCR product was detected in templates prepared from the mutant pool alone or wild-type *Histoplasma*. A smaller PCR amplicon which is not specific to the *ags1*::T-DNA template was detected in all reactions derived from the random insertion mutant pool.

### Nested PCR to reduce false-positives

To discriminate between true- and false-positive PCR products, we employed a secondary PCR reaction using a set of nested primers. Nested primers that do not overlap with the primary PCR primers were designed for both the T-DNA anchor and the *AGS1 *gene. Primary PCR reactions in which OSU4 represented 1/200^th ^or 1/800^th ^of the population were used as templates after 1:1000, 1:10,000, and 1:100,000 dilution in H_2_O. As shown in Figure 1C, this process eliminated the false-positive band observed in the primary PCR reactions. The *ags1*::T-DNA specific amplicon could be detected after either 1:1000 or 1:10,000 dilution of the primary PCR reaction. No *ags1*::T-DNA amplicon was produced when OSU4 was absent in the primary reaction template DNA. These data demonstrate that PCR can be an efficient screening technique to probe mutant pools for a clone in which a T-DNA element has inserted into a target gene. We selected a target pool size of approximately 200 insertion mutants as a balance between increased throughput afforded by larger pools but easier subdivision of smaller pools into individual clones to recover the detected mutant strain (see below).

### Establishment of a bank of insertion mutants

#### Optimization of freezing conditions

**Figure 2:**
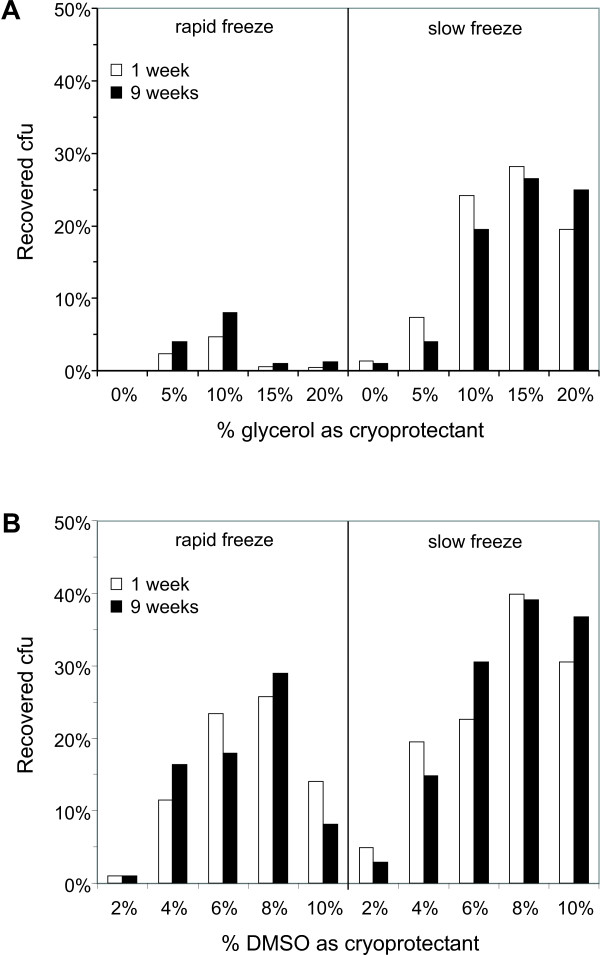
**Gradual freezing in DMSO maximizes recovery of cryopreserved *Histoplasma *yeast**. WU15 yeast were frozen in varying concentrations of glycerol **(A) **or DMSO **(B)**. *Histoplasma *yeast were grown to late log/early stationary phase in rich medium and added to the appropriate glycerol- or DMSO-containing solutions before freezing. Final cryoprotectant concentrations indicated along the x-axis of each graph. Vials were placed immediately at -80°C (rapid freeze) or were placed into a styrofoam container before placement at -80°C (slow freeze). Frozen cell aliquots were thawed after 1 week or 9 weeks and recovery measured as the number of viable cfu relative to the number present before freezing.

As the generation of T-DNA insertion mutants in *Histoplasma *is not trivial, establishment of a frozen bank of insertion mutants would facilitate future screens without having to produce new mutant pools as additional target genes are identified. Maintaining the mutant representation in the pool after freezing necessitates efficient recovery of viable cells following thawing. To maximize the recovery of cells after freezing we examined two parameters: the cryoprotectant used and the method of freezing. Glycerol- or DMSO-containing solutions are used for freezing eukaryotic cells as these chemicals reduce membrane-damaging ice crystal formation. We also tested whether slowing the freezing rate using an insulated container also improved recovery from frozen stocks. *Histoplasma *WU15 yeast cells were frozen and stored at -80°C for 7 days or 9 weeks to determine the short and long term storage recovery rates, respectively. Recovered cfu counts were compared to those before freezing. With glycerol as the cryoprotectant, slowing the freezing rate dramatically improved recovery of viable yeast (Figure 2A), probably resulting from the increased time to allow for penetration of glycerol into cells during cooling. DMSO was a superior cryoprotectant than glycerol for *Histoplasma *yeast when present at concentrations from 4% to 10% (Figure 2B). Freezing with DMSO was less sensitive to the rate of freezing but as with glycerol, slower rates of cooling resulted in greater recovery of viable yeast cells (Figure 2B). Nearly 40% of the starting suspension of yeast cells were recovered when cells were slowly frozen in an 8% DMSO-containing solution and this procedure was selected for long term storage of mutant pools. Although specialized cooling apparatuses can be used to control the freezing rate, we found that simple placement of vials of cells within readily and cheaply obtained styrofoam containers (such as those used for shipments of molecular biology enzymes) was sufficient.

#### Generation of mutant pools

Insertion mutants were generated in the NAm 2 *Histoplasma *strain WU15 by co-cultivation of *Agrobacterium tumefaciens *and *Histoplasma *yeast cells. Co-cultures were plated onto filters and *Histoplasma *transformants selected by transferring filters to medium containing hygromycin to which resistance is provided by sequences within the T-DNA element [23]. Transformant yeast cells were collected and suspensions from individual plates combined to create pools derived from 100 to 200 independent mutant colonies. Yeast cell suspensions were diluted into fresh medium and allowed to grow for 24-48 hours. Twenty-four pools were prepared representing roughly 4000 insertion mutants. A portion of each culture was reserved for nucleic acid isolation and the remainder frozen in aliquots and stored at -80°C. Nucleic acids were purified from each pool, diluted to 50 ng/ul, and stored at -20°C until analysis by PCR.

With an estimated 9000-10,000 genes encoded by the *Histoplasma *genome, this collection does not represent the number of insertion mutants required for saturation of the genome. We used two probability functions to estimate the size of the library required for a 95% chance of isolating an insertion in a particular locus in the 40 megabase NAm 2 genome. Both calculations assume no bias in insertion sites. Based on the number of predicted genes, the Poisson approach estimates a library of approximately 30,000 insertions would be required. The single study in which multiple alleles of a single locus were isolated in *Histoplasma *(five *AGS1 *alleles isolated in a screen of 50,000 insertions; [23]) supports the Poisson calculation; five alleles would be the most probable number of alleles based on a 9000 or 10,000 target estimate. However, the *AGS1 *gene is an unusually large target and thus this number is probably an underestimate. An alternative calculation based solely on average gene size is provided by: P = 1-(1-x/G)^n ^where P is the probability of the T-DNA inserting in a given target of size x in a genome of size G with n the total number of T-DNA insertion mutants [35]. Assuming an average gene size of 2000 nucleotides, this calculation estimates a library of nearly 60,000 mutants would be required for a 95% probability of obtaining at least one insertion mutant in any given gene. Such a mutant bank would require 300 pools with an average pool size of 200 and PCR screening could be easily performed using three 96-well plates. Although our current collection of 4000 mutants is inadequate for complete genome coverage, it was sufficient to demonstrate proof-of-concept through identification and recovery of a mutant at the *CBP1 *locus.

### Isolation of a *cbp1 *insertion mutant

#### Detection of a T-DNA insertion in *CBP1*

**Figure 3:**
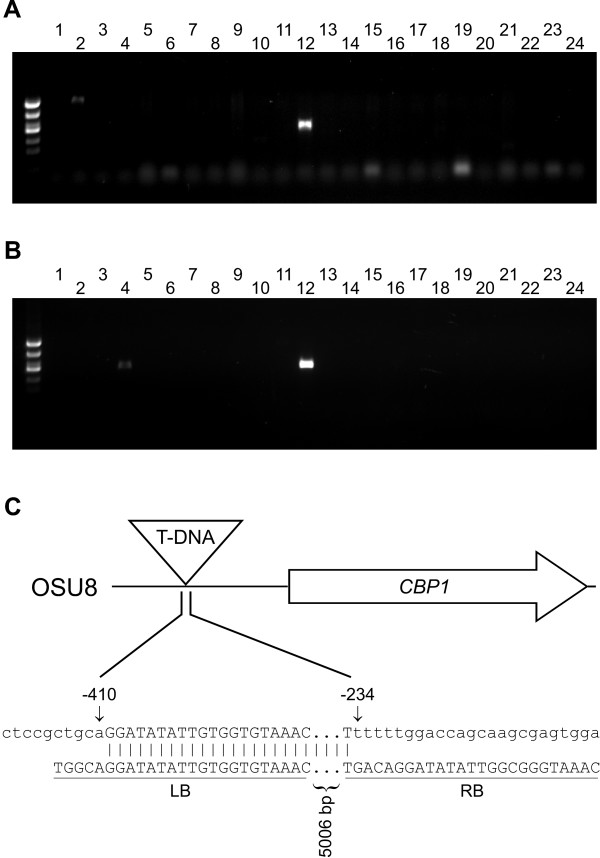
**PCR screening of a mutant pool bank identifies an insertion in the *CBP1 *locus**. Twenty-four pools of T-DNA insertion mutants were screened by primary PCR **(A) **and nested PCR **(B) **with primer sets specific for the *CBP1 *gene. **(A) **Template nucleic acids from 24 mutant pools (each comprised of 100-200 individual mutants) were screened with the RB3 and CBP1-21 primers. The reaction products for each pool were separated in individual lanes by electrophoresis through 1% agarose. **(B) **Primary PCR reactions from (A) were diluted 1:10,000 and used as template for nested PCR with RB6 and CBP1-23 primers. The potential *cbp1*::T-DNA mutant was found only in pool #12. **(C) **Schematic depiction of the identified *cbp1*::T-DNA insertion. The T-DNA insertion from pool #12 was designated OSU8. Sequencing of the PCR product from regions flanking the insertion localized the T-DNA element insertion site 234 base pairs (bp) upstream of the *CBP1 *coding region. Nucleotide sequences flanking the T-DNA insertion in the mutant (top row) aligned with the T-DNA left border (LB) and right border (RB) imperfect direct repeats (bottom row) show the nature of the mutational event. Numbers above the mutant sequence correspond to nucleotides of the wild-type *CBP1 *promoter.

As no *cbp1 *mutant exists in the NAm 2 background despite numerous attempts with allelic replacement, we screened our NAm 2 mutant bank for T-DNA insertions that disrupt the *CBP1 *gene. The Cbp1 protein was the first virulence factor demonstrated for *Histoplasma *through deletion of the encoding gene in a Panama class strain of *Histoplasma *[20]. Two *CBP1 *gene-specific primers were designed at the 3' end of the *CBP1 *coding region and were oriented towards the 5' end of the gene. As the T-DNA element could insert with either the T-DNA left border or the right border oriented towards the 3' end of the *CBP1 *gene, we screened each mutant pool by PCR with RB3 or with LB6 primers in combination with the CBP1-21 gene-specific primer. While PCR reactions with the LB6 + CBP1-21 primer set did not produce any positive PCR products with any of the templates (data not shown), reactions with RB3 and CBP1-21 primers produced amplicons in two different pools (Figure 3A, lanes 2 and 12). Low abundance bands less than 100 bp are likely primer dimers or residual RNA from the template nucleic acids and were thus not considered. A nested PCR reaction was performed on the RB3-set of reactions (Figure 3B). The PCR product from pool 2 did not re-amplify in the nested PCR reaction suggesting that this product was a non-specific amplicon. Alternatively, the pool may indeed harbor an insertion of T-DNA sequence in the *CBP1 *locus but the T-DNA element could be truncated and the nested RB primer-binding site lost resulting in failure to amplify in the nested PCR. The nested PCR reaction from pool 12 produced a very prominent, approximately 800 bp amplicon consistent with an insertion in the DNA upstream of the *CBP1 *coding region (Figure 3B, lane 12). Sequencing of this amplicon confirmed insertion of the T-DNA in the *CBP1 *promoter and localized the insertion 234 base pairs upstream of the *CBP1 *start codon (Figure 3C). Although a faint band was observed in the secondary PCR from pool #4, no product was detected in the primary reaction and it's abundance was much lower than that of the validated amplicon from pool #12. Thus, it was discarded as a candidate.

#### Recovery of the *cbp1 *insertion mutant

**Figure 4:**
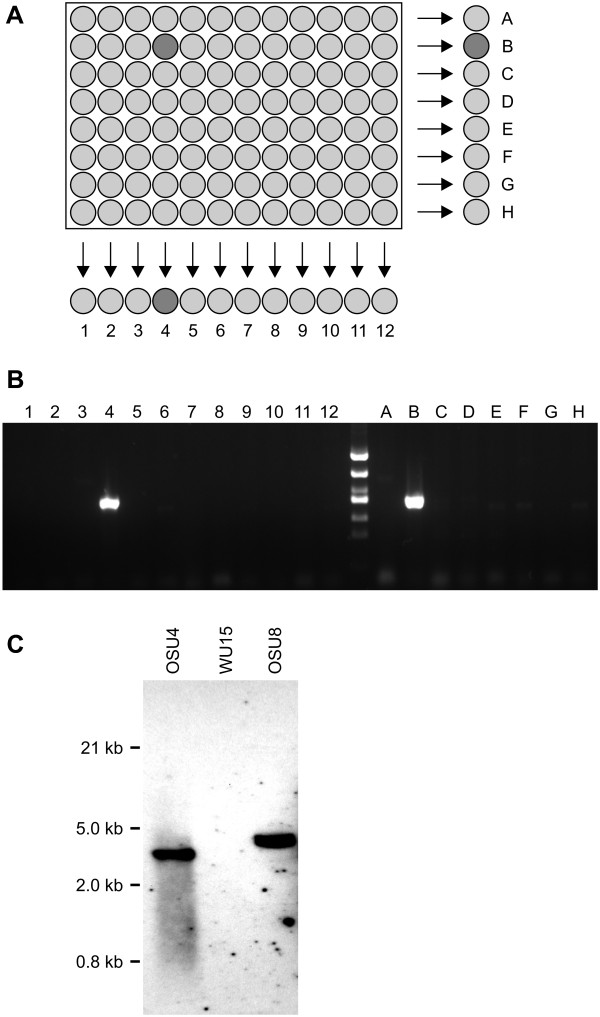
**Recovery of the *cbp1 *mutant from mutant pool 12**. **(A) **Diagram showing the addressing strategy used to efficiently identify which of 96 constituents of pool 12 correspond to the targeted *cbp1 *mutant. Individual clones were arrayed into 96-well plates and sub-pools created representing each row (letters) and column (numbers). Shaded wells depict the desired *cbp1*::T-DNA insertion clone or row and column sub-pools containing the clone. **(B) **Identification of the clone corresponding to the *cbp1*::T-DNA mutant. PCR was performed on each column and row sub-pool with the RB6 and CBP1-23 primers. Positive PCR amplicons identified the isolate at B4 as the *cbp1*::T-DNA mutant. **(C) **Southern blot analysis of the mutant strains with T-DNA insertions. Hind III-digested genomic DNAs prepared from OSU4, WU15, and OSU8 strains were probed with a T-DNA-specific probe. Single 3.8 kb and 3.0 kb bands detected in OSU4 and OSU8, respectively, indicate the mutant strains do not harbor multiple integrations of the T-DNA element.

To isolate the strain containing the *cbp1*::T-DNA mutation, we recovered yeast cells from pool #12 and segregated the pool into individual clones. The insertion was tracked using PCR with the primers described earlier. Pool #12 was thawed and dilutions plated to recover individual cfu's. As each pool represents 100-200 clones, we screened 286 clones to increase the likelihood of recovering at least one strain with the detected *CBP1 *insertion mutation. To drastically reduce the number of nucleic acid preparations and PCR tests required to screen nearly 300 individuals, we employed an addressing scheme (schematically shown in Figure 4A). Each clone was picked into individual wells of three 96-well plates containing liquid medium. For each 96-well plate, wells from each row and from each column were pooled to produce 20 yeast suspensions. An aliquot of these row and column sub-pools from each plate were combined to create a yeast suspension representing the clones from the entire 96-well plate. Nucleic acids were isolated from the three 96-well plate suspensions and subjected to PCR. The *cbp1*::T-DNA insertion amplicon was detected in two of the three collections of 96 (data not shown). For one of these pools of 96 individual clones, nucleic acids were isolated from the corresponding row and column sub-pools and PCR was used to screen for the T-DNA insertion. Positive amplicons were detected in sub-pools representing the clone located at B4 (Figure 4B). The suspension of yeast was recovered from well B4 and plated on solid medium to recover individual colonies. PCR performed on nucleic acids prepared from one of these colonies confirmed the identity and recovery of the desired insertion at the *CBP1 *locus. The strain with this insertion was designated OSU8.

To further characterize the T-DNA insertion in OSU8, we amplified and sequenced the DNA flanking the T-DNA element. PCR amplicons were produced for both the left and right border flanking regions using T-DNA specific primers and *CBP1 *specific primers (data not shown). Alignment of the flanking regions with the *Histoplasma *G217B genome and T-DNA sequences showed truncation of the T-DNA imperfect direct repeats by 5 bp from the left border and 24 bp from the right border. Additionally, the T-DNA insertion event deleted 175 base pairs of the *CBP1 *promoter surrounding the site of insertion (Figure 3C). Due to T-DNA-induced genetic rearrangements that can occur, PCR-product sizes should be used only as an initial estimate of the location of T-DNA integration and the precise location of the insertion confirmed by sequencing the DNA flanking the T-DNA element. As our PCR screening method would not detect multiple T-DNA integrations, we performed a Southern blot using a T-DNA-specific probe to determine how many T-DNA elements were present in the OSU8 mutagenized genome. As shown in Figure 3D, only one band is detected indicating the OSU8 strain harbors a single T-DNA insertion. This 3.8 kb T-DNA probe-hybridizing fragment is the size predicted for the described insertion in the *CBP1 *promoter. No T-DNA sequences were detected in the parental WU15 strain.

#### Validation of the *cbp1 *mutant

**Figure 5:**
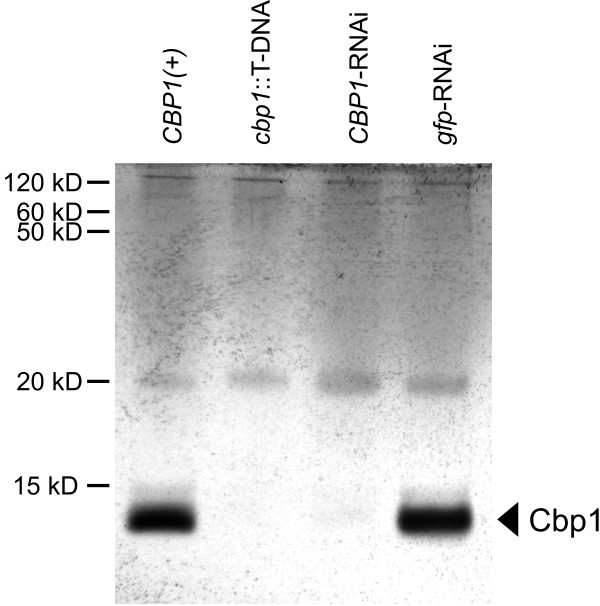
**The T-DNA insertion in *CBP1 *prevents production of the Cbp1 protein**. Culture supernatants from the *cbp1*::T-DNA insertion (OSU8) lack the Cbp1 protein whereas culture supernatants from CBP1(+) yeast cells (WU15) show abundant production of Cbp1. Cell-free culture supernatants were prepared from late log/early stationary phase cultures of *Histoplasma *yeast and the major secreted proteins separated by electrophoresis. The Cbp1 protein runs as a 9-11 kD band. Positive identification of this band as Cbp1 was determined by loss of the 9-11 kD protein band from supernatants derived from a *CBP1*-RNAi strain (OSU38). A strain harboring a *gfp*-RNAi plasmid (OSU37) was used to show specific depletion of Cbp1 by *CBP1*-RNAi in OSU38. The secreted 20 kD protein produced by all strains was used to normalize supernatant loadings.

Since the T-DNA insertion in OSU8 did not lie within the *CBP1 *gene but was instead located in the sequence upstream of the *CBP1 *coding sequence, we tested whether the recovered mutant had lost the ability to produce the Cbp1 protein. The T-DNA insertion site lies within the region defined as the *CBP1 *promoter [36]; transcriptional fusion constructs with less than 689 base pairs of sequence upstream of *CBP1 *do not support transcription. Thus, insertion of 5 kb of foreign sequence (i.e. the T-DNA element) into this region should disrupt promoter activity. OSU8 and the parent WU15 strain were grown to early stationary phase and cell-free supernatants were prepared. To determine whether Cbp1 production was impaired in OSU8, we separated supernatant proteins by poly-acrylamide gel electrophoresis and visualized the proteins by silver staining. Supernatants from the *CBP1*(+) WU15 strain had a prominent 9-11 kD protein which was not detected in supernatants harvested from the OSU8 culture (Figure 5) indicating the *cbp1*::T-DNA insertion disrupts production of Cbp1 protein. The identity of this protein was confirmed as Cbp1 since supernatant from a strain in which Cbp1 was independently depleted by RNAi also specifically lacked this protein band. Thus, while the T-DNA insertion does not interrupt the coding region, insertion into the *CBP1 *promoter effectively prevents production of Cbp1 in OSU8.

## Conclusion

We have developed a reverse genetics procedure employing random mutagenesis and PCR-based screening techniques to identify insertion mutants in a targeted gene in *Histoplasma capsulatum *without regard to a mutant phenotype. Since the mutagen creates a large insertion, the majority of mutations should reflect the knock-out mutant phenotype. However, insertions within the promoter of a gene may allow some residual transcription resulting in hypomorphic but not null phenotypes. In such cases, as demonstrated by our *cbp1*:T-DNA mutant, delineation of the minimal promoter of a targeted gene could resolve what type of phenotype the insertion mutation would likely produce. Thus, the regions most likely to produce mutant phenotypes are the proximal promoter and the coding region of the targeted gene. Consequently, we routinely design our PCR screening primers at the 3' end of the gene to amplify these regions in particular and maximize the targeted site for insertions. This process, as with any mutational approach, will not successfully isolate insertion mutations in genes essential for viability, and if the intended target is suspect to be such, RNAi may prove more beneficial. While integration of T-DNA into the *Histoplasma *genome appears relatively random, large scale studies in *Magnaporthe*, *Leptosphaeria*, and *Arabidopsis *indicate there is a bias for insertion of the T-DNA element into non-coding regions [37-40]. In addition, occurrence of large-scale deletions or rearrangement mutations will be missed by this approach. Thus, more insertion mutants may be required for saturation mutagenesis of the *Histoplasma *genome than calculated above.

The reverse genetics process detailed here increases the repertoire of methods available to disrupt gene functions in *Histoplasma capsulatum*. Since *Agrobacterium*-mediated transformation has been developed as an efficient mutagen for a variety of fungal species [41], this procedure should be readily applicable to those microorganisms as well. For intractable fungal systems where homologous recombination is very limited or allelic replacement unfeasible, this process provides the ability to disrupt gene functions necessary for functional genetic tests. The only requirement is an efficient insertional mutagen. The increased capability to disrupt gene functions in *Histoplasma *and in other fungi will greatly improve our mechanistic understanding of fungal biology.

## Methods

### Yeast strains and culture

All experiments were performed with strains derived from the clinical NAm 2 *Histoplasma capsulatum *isolate G217B (ATCC 26032) and are listed in Table 1. WU15 is a uracil auxotroph due to mutation of the *URA5 *gene [23]. OSU4 was derived from WU15 by *Agrobacterium*-mediated transformation and harbors a T-DNA insertion in the *AGS1 *gene. *Histoplasma capsulatum *was grown in HMM medium at 37°C with 5% CO_2_/95% air with shaking (200 rpm) as previously described [42]. For platings, HMM was solidified with 0.6% agarose (USB) and 25 uM FeSO_4 _was added. HMM was supplemented with uracil (100 ug/ml) for growth of uracil auxotrophs and hygromycin B (200 ug/ml) for selection of T-DNA insertion mutants.

**Table 1:** *Histoplasma *strains

strain	genotype
WU15	*(G217B) ura5-Δ42*
OSU4	*(G217B) ura5-Δ42 ags1-5::T-DNA [hph]*
OSU8	*(G217B) ura5-Δ42 cbp1-9::T-DNA [hph]*
OSU37	*(G217B) ura5-Δ42/pCR473 [URA5, gfp-RNAi]*
OSU38	*(G217B) ura5-Δ42/pCR475 [URA5, CBP1-RNAi]*

### *Agrobacterium*-mediated transformation of *Histoplasma*

*Agrobacterium tumefaciens *was used to transform *Histoplasma capsulatum *yeast using modifications to previously described protocols [23,31]. *A. tumefaciens *strain LBA1100 was transformed with pCM41, an engineered plasmid containing a hygromycin resistance cassette flanked by the left and right border T-DNA sequences [23]. *A. tumefaciens *harboring pCM41 was grown in LC media [43] containing 100 ug/ml kanamycin and 250 ug/ml spectinomycin to select for the T-DNA and Ti plasmids, respectively. Liquid LC media was inoculated with 10 colonies and grown overnight at 25°C with shaking (250 rpm). Bacteria were harvested by centrifugation (5000 × g) and resuspended in 5 volumes *Agrobacterium *induction medium [IM-- 44] and grown for approximately 16 hours at 25°C with shaking. Complete induction medium contained 0.2% glucose, antibiotics, and 200 uM acetosyringone and was buffered to pH 5.3 with MES. Bacteria were collected by centrifugation and resuspended in induction medium to an optical density at 600 nm of 1.5 (corresponding to roughly 1.5 × 10^9 ^bacteria/ml). *Histoplasma *WU15 yeast were harvested from solid HMM + uracil medium seeded 3 days earlier with 4 × 10^5 ^yeast/cm^2^. Yeast were collected by flooding plates with 5 mls HMM medium and scraping with a sterile spreader. Yeast were collected by centrifugation (1000 × g) and resuspended in induction medium at a density of 5 × 10^8 ^yeast/ml as determined by hemacytometer counts. For co-cultivation, 1.5 × 10^8 ^*Agrobacterium *cells were mixed with 5 × 10^7 ^*Histoplasma *yeast in a total volume of 400 ul and spread on Whatman #5 filter paper placed on top of solid induction medium supplemented with 0.7 mM cystine and 100 ug/ml uracil. Plates were incubated for 48 hrs at 25°C after which filters were transferred to selection medium (HMM + uracil + hygromycin + 200 uM cefotaxime) and incubated at 37°C with 5% CO_2_/95% air until *Histoplasma *transformants became visible (10-14 days). Six cm diameter plates were used so that roughly 50-100 transformants were obtained per plate.

### PCR-based screening of T-DNA insertion mutants

Hygromycin-resistant transformants of *Histoplasma *were collected by flooding plates with HMM and suspending cells with a sterile spreader. Suspensions from individual plates were combined to obtain pools representing 100-200 independent transformant colonies. Yeast suspensions were diluted 1:10 into 10 mls HMM + uracil and grown for 24-48 hours. Two milliliters of culture were collected for nucleic acid isolation and the remaining culture frozen in 1 ml aliquots for later recovery of yeast.

To purify *Histoplasma *nucleic acid for PCR, cells were collected by centrifugation (2000 × g) and nucleic acids released by mechanical disruption of yeast in the presence of detergents and organic solvent [45]. 250 ul of lysis buffer (20 mM Tris pH 8.0, 200 mM NaCl, 2 mM EDTA, 2% SDS, 4% Triton X-100) and 250 ul of phenol:chloroform:isoamyl alcohol (25:24:1) were added to cells and nucleic acids released by bead beating cells with 0.5 mm-diameter acid-washed glass beads. Phases were separated by centrifugation (5 minutes at 14,000 × g) and the aqueous phase transferred to new tubes. Nucleic acids were recovered by precipitation of the aqueous phase with 2.5 volumes of ethanol. As no efforts were taken to remove RNA co-purifying with the DNA, total nucleic acids were quantified by spectrophotometric readings at 260 nm

Screening of pools was done by two sequential PCR steps. Primers used are listed in Table 2. For the primary PCR, 50 ng of total nucleic acid was used as template in a 25 ul reaction with either a left border (e.g., LB6) or right border primer (e.g., RB3) and a primer specific for the gene of interest. 30 cycles of PCR were performed and the reaction diluted 1:10,000 before use as template in a nested PCR reaction employing a gene specific primer in conjunction with a nested left border or right border primer (LB8 or RB6, respectively). Thirty cycles were performed for the nested PCR followed by electrophoretic separation of products in 1% agarose gels. The following program was used for amplification reactions: 2 min at 94°C; 30 cycles of 10 seconds at 94°C, 15 seconds at 54°C, and 2 minutes at 72°C. Amplifications used Taq polymerase (Invitrogen). Pools yielding PCR products were confirmed by repeating the PCR with single primer controls as well as the combined primer set.

**Table 2:** Oligonucleotides used for screening T-DNA insertion pools

	sequence	T_**m**_^**1**^
T-DNA primers		
RB3	CGAATTCGAGCTCGGTACAGTGAC	58°C
RB6	GATTGTCGTTTCCCGCCTTCAG	59°C
LB6	TGTTGGACTGACGCAACGACCTTGTCAACC	69°C
LB8	CAGGGACTGAGGGACCTCAGCAGGTCG	68°C
		
Gene-specific primers		
AGS1-50	ATCCATCATTCAACGTCCGGTA	56°C
AGS1-72	TTGCGTACTGGGTGAGATGG	54°C
CBP1-21	AATCACGTGGTCGCTAAATGG	54°C
CBP1-23	CCACAAGCAGCCCTTGCATGCCTCA	67°C

^1 ^T_m _calculated annealing temperature

### Addressing and recovery of T-DNA insertion mutants

Yeast from pools showing a positive PCR were thawed from frozen stocks and dilutions were plated on solid HMM + uracil medium to obtain individual clones. One millimeter-diameter colonies were individually picked into 150 ul of HMM + uracil medium in 96-well plates and 25 ul from the wells of each row and column pooled using a multi-channel pipettor. The remaining yeast suspension in the 96-well plate was grown at 37°C with 5% CO_2_/95% air while addressing PCR was performed. Nucleic acids were prepared from the row and column pools and used as template for PCR. Yeast were recovered from positive wells and plated on solid medium. Single clones were isolated and template nucleic acids prepared for use in PCR. PCR amplicons were purified and sequenced to confirm and localize the insertion in the gene of interest.

### Southern blot analysis of T-DNA insertion mutants

T-DNA mutant and WU15 genomic DNAs were prepared and digested overnight with Hind III. Nucleic acid fragments were separated by agarose gel electrophoresis and transferred to a Nytran membrane using a vacuum blot apparatus. Fragments were fixed to the membrane by ultraviolet irradiation (254 nm wavelength, 120,000 uJ/cm^2^; Stratalinker UV Crosslinker, Stratagene). A nucleic acid probe from the right side of the T-DNA element was prepared by PCR and labeled using the AlkPhos Direct Labeling System (Amersham). The T-DNA probe was hybridized to the membrane and was detected by chemiluminescence using the CDP-Star reagent (Amersham).

### Cryopreservation of *Histoplasma *yeast

*Histoplasma *yeast were collected from exponential or early stationary phase cultures and added to vials containing either glycerol or dimethylsulfoxide (DMSO). For rapid freezing, vials were mixed and then placed immediately in a rack at -80°C. For gradual freezing, vials were placed within a styrofoam container which was then placed at -80°C. After 24 hours, vials were transferred to racks and stored at -80°C. For recovery, vials were thawed by incubation in a 37°C water bath followed by addition of 2 volumes 37°C HMM. Serial 5-fold dilutions were plated on solid HMM + uracil medium to enumerate viable colony forming units (cfu) for each freezing condition and results were compared to cfu counts before freezing.

### Cbp1 production assay

*Histoplasma *yeast were grown in liquid HMM media to an optical density at 595 nm of 3.2 - 3.8. *Histoplasma *yeast were removed by centrifugation for 5 minutes at 2000 × g. The supernatant was further clarified by centrifugation for 5 minutes at 15,000 × g. SDS- and DTT-containing protein sample buffer was added to culture supernatants and the proteins separated by 12% poly-acrylamide gel electrophoresis using a Tris-tricine buffer system. The major culture filtrate proteins were visualized by silver staining of gels.

## Authors' contributions

BY performed the mutant pooling, screening and optimization, and recovery of insertion mutants. JD participated in the screening and recovery. CR performed the mutagenesis and freezing condition optimization. CR conceived the study and coordinated its design and execution. BY and CR drafted the manuscript. All authors read and approved the final manuscript.
